# *Haloglomus irregulare* gen. nov., sp. nov., a New Halophilic Archaeon Isolated from a Marine Saltern

**DOI:** 10.3390/microorganisms8020206

**Published:** 2020-02-02

**Authors:** Ana Durán-Viseras, Cristina Sánchez-Porro, Antonio Ventosa

**Affiliations:** Department of Microbiology and Parasitology, Faculty of Pharmacy, University of Sevilla, 41012 Sevilla, Spain; anaduran@us.es (A.D.-V.); sanpor@us.es (C.S.-P.)

**Keywords:** haloarchaea, hypersaline habitats, salterns, taxonomy, ecology, *Haloglomus*

## Abstract

A halophilic archaeal strain, designated F16-60^T^, was isolated from Isla Cristina marine saltern in Huelva, Spain. Cells were pleomorphic, irregular, non-motile, and Gram-stain-negative. It produced red-pigmented colonies on agar plates. Strain F16-60^T^ was extremely halophilic (optimum at 30% (*w*/*v*) NaCl) and neutrophilic (optimum pH 7.5). Phylogenetic tree reconstructions based on 16S rRNA and *rpoB´* gene sequences revealed that strain F16-60^T^ was distinct from species of the related genera *Natronomonas*, *Halomarina*, and *Halomicrobium*, of the order *Halobacteriales*. The polar lipids are phosphatidylglycerol (PG), phosphatidylglycerol phosphate methyl ester (PGP-Me), phosphatidylglycerol sulfate (PGS), and one glycolipid chromatographically identical to sulfated mannosyl glucosyl diether (S-DGD-1). The DNA G+C content is 68.0 mol%. The taxonomic study, based on a combination of phylogenetic, genomic, chemotaxonomic, and phenotypic analyses, suggest that strain F16-60^T^ (= CECT 9635^T^ = JCM 33318^T^), represents a novel species of a new genus within the family *Haloarculaceae* and the order *Halobacteriales*, for which the name *Haloglomus irregulare* gen. nov., sp. nov. is proposed. Metagenomic fragment recruitment analysis revealed the worldwide distribution of members of this genus and suggested the existence of other closely related species to be isolated.

## 1. Introduction

The class *Halobacteria* is a major group within the domain *Archaea*, and comprises halophilic archaea (also called haloarchaea) which are typically found in hypersaline environments, such as salt lakes, soda lakes, or salterns [1]. The class *Halobacteria* includes three orders, *Haloferacales*, *Halobacteriales*, and *Natrialbales*, which consist of several different families [2,3,4]. Haloarchaea exhibit enormous diversity in terms of their morphology or physiology. They can be coccoid, rod-shaped, pleomorphic, or even square, motile, or non-motile cells. In addition to their extreme NaCl requirements, they may grow at neutral or alkaline conditions, and thus they may be haloalkaliphiles [1,4,5].

Marine salterns are thalassohaline environments, which constitute excellent models for ecological studies based on their structure of a series of ponds with different salinities, and thus, have been long-term targets for the study of halophilic microbial diversity [6]. One of these salterns is located in Isla Cristina, Southwest coast of Spain (37° 12′ N 7° 19′ O). Metagenomic studies carried out in an intermediate salinity pond (21% salts) of this saltern showed the predominance of members of the phylum *Euryarchaeota*, followed by the phylum *Bacteroidetes*, being the genera *Halorubrum*, *Psychroflexus*, and *Natronomonas* the most abundant groups. Moreover this analysis also brought to light that a large number of haloarchaeal members have not been isolated until date [7,8]. On the other hand, several culture-dependent studies performed in these salterns have permitted the isolation and characterization of new groups of halophilic bacteria, such as members of the genera *Spiribacter* [9,10], *Idiomarina* [11], *Marinobacter* [12], or *Salinivibrio* [13], as well as of the archaeal genera *Halonotius* [14,15] and *Halorientalis* [16]. In 2016, as part of a new study of the halophilic archaeal diversity of Isla Cristina marine saltern, we isolated a halophilic archaeal strain, designated F16-60^T^, closely related to members of the order *Halobacteriales*. In the present study, we describe the properties of strain F16-60^T^ on the basis of recommended standard taxonomic methods, as well as on recent genomic approaches, following the respective minimal standards [17,18]. Our data suggest that strain F16-60^T^ is not related to any previous haloarchaeal taxa and thus, we propose it as a new genus and species, *Haloglomus irregulare* gen. nov., sp. nov. In addition, we determined that this new haloarchaeon is widely distributed in different hypersaline habitats.

## 2. Materials and Methods

### 2.1. Isolation of Haloarchaeal Strain

During the course of studies on Isla Cristina saltern, Southwest coast of Spain (37° 12´ 30´´–7° 19´ 41´´) in June 2016, a halophilic archaeal strain, designated F16-60^T^, was isolated from saline water of a pond of the saltern. At the time of sampling, the salinity of the pond was 32% (*w*/*v*) total salts and the pH was 7.5. Samples were transported to the laboratory in a short time and plated under sterile conditions. Plates were incubated at 37 °C for up to three months. Strain F16-60^T^ was isolated in pure culture after successive cultivations, using a medium prepared by filtering the saline water from the pond, with no other nutrients added, and with 2% (*w*/*v*) purified agar (Oxoid) as solidifying agent. The pH of the medium was adjusted to 7.5 with 1 M KOH. Strain F16-60^T^ was routinely grown in the modified DBCM2 medium (CECT medium no. 263) and incubated aerobically at 37 °C, using a rotary shaker for growth in liquid medium. The composition of the modified DBCM2 medium is: sodium pyruvate 0.011 g, glucose 0.00025 g, peptone (Difco) 0.00125 g, yeast extract (Difco) 0.00125 g, distilled water 16.67 ml, and 83.33 ml of a stock of seawater SW30 solution, which contained (g/L): NaCl: 195; MgCl_2_.6H_2_O: 32.5; MgSO_4_.7H_2_O: 50.8; CaCl_2_: 0.83; KCl: 5.0; NaHCO_3_: 0.17; NaBr: 0.58. Purified agar 2% (*w*/*v*) (Oxoid) was added when necessary. For long time preservation, cultures were maintained at −80 °C in a liquid medium containing 40% (*v*/*v*) glycerol. Halophilic archaeal type strains *Natronomonas pharaonis* CECT 4578^T^ and *Natronomonas moolapensis* CECT 7526^T^, obtained from culture collections, were also used in this study as reference strains. 

### 2.2. DNA Extraction, Purification, and Sequencing

DNA extraction for determining the 16S rRNA gene sequence, *rpoB´* gene sequence and genome sequence analysis, was obtained using the Marmur method [19]. The quantification of the extracted DNA was determined by spectrophotometry (DeNovix DS-11 FX, DeNovix Technologies, Wilmington, DA, USA) and fluorometry (Qubit 3.0 Fluorometer, Thermofisher Scientific, Waltham, MA, USA). DNA quality was checked by (1%) agarose gel electrophoresis. The 16S rRNA gene and the *rpoB´* gene were amplified by PCR [20] using the universal primers for archaea ArchF and ArchR [21,22] and the primers designed by Fullmer et al. [23], respectively. Sequencing of PCR products were carried out by StabVida (Oeiras, Portugal) using the Sanger method. The GenBank/EMBL/DDBJ accession number for the 16S rRNA and *rpoB´* gene sequences of strain F16-60^T^ are MH424600 and MK182272, respectively. The genome of strain F16-60^T^ was sequenced using the lllumina Hiseq 4000 platform (StabVida, Oeiras, Portugal).

### 2.3. Genome Assembly and Annotation

The de novo assembly of the reads was performed using Spades 3.9.1 [24]. The quality of final contigs was assessed by the bioinformatics tool CheckM v1.0.5 [25] and Quast v2.3 [26]. The genome sequence was annotated using the NCBI Prokaryotic Genome Annotation Pipeline (PGAP) [27]. The genome of strain F16-60^T^ was deposited in GenBank/EMBL/DDBJ under the accession number QMDX00000000.

### 2.4. Phylogenetic Analyses

The identification of phylogenetic neighbours and calculation of the pairwaise 16S rRNA gene sequence similarity of strain F16-60^T^ was carried out by using the EzBioCloud server [28] and GenBank. The 16S rRNA and the *rpoB´* gene sequences used for phylogenetic comparisons were obtained from GenBank. To delineate the phylogenetic position, gene sequences alignments were performed using ChromasPro (Technelysium Pty Ltd, Tewantin, Australia) software version 1.5. Bootstrap consensus trees were inferred from 1000 replicates [29] using the neighbour-joining [30], maximum-parsimony [31], and maximum-likelihood [32] algorithms integrated in the ARB software package [33] for the 16S rRNA gene phylogenetic analysis and with MEGA 6.0 software [34] for the *rpoB´* gene analysis. Evolutionary distances were calculated according to the algorithms of the Jukes–Cantor model [35]. To calculate phylogenomic relations between strain F16-60^T^ and phylogenetic neighbours, a core-genome tree was constructed. Available genomic sequences of the closely related taxa were also obtained from GenBank. All predicted protein-coding genes annotated from each available genome were compared using the all-versus-all BLAST search [36]. MUSCLE [37] was used for the alignment of core orthologous genes. MEGA 6.0 software [34] was used for the phylogenomic tree reconstruction, by the neighbour-joining method with Jukes–Cantor correction [35].

The number of common genes shared between strain F16-60^T^ and phylogenetic neighbours were represented by a Venn Diagram, which was constructed using the online software InteractiVenn [38].

### 2.5. Average Nucleotide Identity (ANI), Average Amino Acid Identity (AAI), and Digital DNA–DNA Hybridization (DDH)

To confirm the status as a new taxon of the isolated strain, Average Nucleotide Identity (ANI), Average Amino Acid Identity (AAI), and in silico DNA–DNA hybridization (DDH) were performed. To calculate ANI, the OAT software v0.93.1 was used [39], while for the estimation of AAI we used the tool AAI calculator [40]. The in silico DDH percentages were calculated by the Genome-to-Genome Distance Calculator (GGDC) [41] website, using formula 2 [42].

### 2.6. Chemotaxonomic Analysis

To complete the chemotaxonomic characterization, strain F16-60^T^ was grown in a modified DBCM2 liquid medium and incubated for three weeks to obtain cell biomass. *Natronomonas pharaonis* CECT 4578^T^, *Natronomonas moolapensis* CECT 7526^T^, *Halobacterium salinarum* DSM 3754^T^, and *Halorubrum saccharovorum* DSM 1137^T^ were used as reference species for polar lipids characterization. Polar lipids were extracted with chloroform/methanol according to Corcelli and Lobasso [43]. Analysis of polar lipids was carried out by one-dimensional HPTLC on Merck silica gel plates crystal back (Merck art. 5626; 10 × 20 cm). Plates were eluted in the solvent system chloroform/90% methanol/acetic acid (65:4:35, by vol.) [44,45]. To detect all lipids, the plate was sprayed with sulfuric acid 5% in water and charred by heating at 160 °C; for specific phospholipids detection, the plate was sprayed with a molybdenum blue spray reagent [46].

### 2.7. Phenotypic Characterization

All morphological and physiological characteristics of strain F16-60^T^ were determined according to the minimal standards for description of new taxa in the *Halobacteria* [17]. The range of NaCl concentrations for growth was evaluated using a modified DBCM2 medium at 0%, 3%, 5%, 10%, 15%, 20%, 25%, 30%, and 35% (*w*/*v*) NaCl concentrations. The range of pH for growth was determined at 5.0, 6.0, 6.5, 7.0, 7.5, 8.0, 8.5, 9.0, or 10.0 and the range of temperature from 20 to 55 °C in 5 °C intervals. Gram staining was performed using acetic acid-fixed samples [47]. Cell morphology and motility were examined in a liquid medium after three weeks of growth by the hanging drop method using an Olympus BX41 phase-contrast microscope. The anaerobic growth was determined using an anaerobic jar with an anaerobic gas generator and anaerobic indicator (Oxoid). Catalase activity was assessed by adding a 3% (*w*/*v*) H_2_O_2_ solution to colonies on a solid medium [48]. Oxidase activity was examined by using 1% (*v*/*v*) tetramethyl-p-phenylenediamine [49]. A test for the hydrolisis of Tween 80, gelatin, and DNA were carried out as described by Barrow and Feltham [48]. The indole production from tryptophan and urease test were assessed as described by Gerhardt et al. [50]. The production of H_2_S was performed according to Clarke [51]. The methyl red and Voges–Proskauer tests, Simmons´s citrate, and anaerobic growth in the presence of nitrate, arginine, or DMSO were evaluated following the minimal standards [17]. The reduction of nitrate and nitrite were detected by using sulfanilic acid and α-naphthylamine reagents [52]. The production of acid from different carbohydrates was tested in a medium with 0.5% (*w*/*v*) yeast extract supplemented with 1% (*w*/*v*) of the carbohydrate tested [17]. To determine the utilization of different organic substrates as carbon and energy sources, a medium containing 0.05% (*w*/*v*) yeast extract supplemented with 1% (*w*/*v*) of the tested substrate was used [53]. The halophilic archaeon *Natronomonas moolapensis* CECT 7526^T^ was used in this study for comparison as a reference strain. *Natronomonas pharaonis* CECT 4578^T^, the type species of the genus, due to its alkaliphilic character, does not grow in the non-alkaliphilic media used for the characterization of strain F16-60^T^, and thus was not used as a reference strain in these studies of phenotypic characterization.

### 2.8. Metagenomic Fragment Recruitment Analysis

To evaluate the presence in hypersaline habitats of haloarchaeal strains related (at species level) to strain F16-60^T^, fragment recruitments with environmental datasets (Table S1) were performed. Genome contigs were concatenated and all the rRNA gene sequences present were masked. Blastn (with the cut-offs: Alignment length ≥ 30 nt, identity > 95%, E-value  ≤ 1  × 10^−5^) was used in order to align the metagenomic quality-filtered shotgun reads (Table S1) against strain F16-60^T^ genome. Best-hits blastn results obtained were used to construct the figures.

## 3. Results and Discussion

### 3.1. Phylogenetic Analyses

After extensive haloarchaeal strains isolations using different media and culture conditions, strain F16-60^T^ was isolated as indicated previously, obtained in pure culture and selected for further analyses.

**Figure 1:**
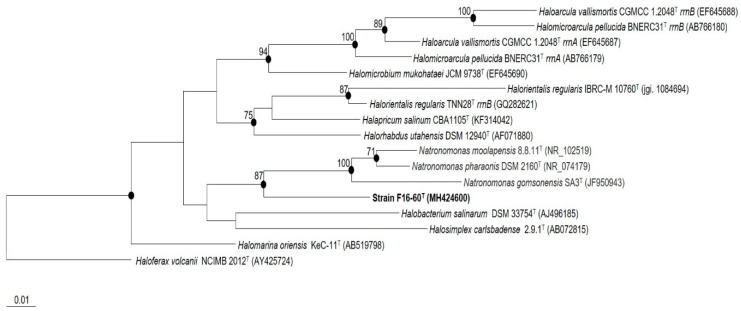
Maximum-likelihood phylogenetic tree based on 16S rRNA gene sequence of strain F16-60^T^ and related members of the order *Halobacteriales*. Filled circles indicate branches that were supported by the neighbour-joining, maximum-parsimony, and maximum-likelihood algorithms. Bootstrap values higher than 70% are indicated at branch-points. Sequence accession numbers are shown in parentheses. Bar: 0.01 substitutions per nucleotide position. The species *Haloferax volcanii* NCIMB 2012^T^ was used as an outgroup.

The EzBioCloud tool was used for the 16S rRNA gene sequence comparative analysis and showed that strain F16-60^T^ (1400 bp) shared the higher sequence similarities with the type strains *Natronomonas moolapensis* 8.8.11^T^ (92.8% 16S rRNA gene sequence similarity) and *Natronomonas pharaonis* DSM 2160^T^ (92.6% 16S rRNA gene sequence similarity), followed by species of other genera such as *Halobacterium*, *Halomarina*, and *Halomicrobium*, with percentages of 16S rRNA gene sequence similarity in all cases lower than 92.5%. The 16S rRNA phylogenetic analysis, based on the maximum-likelihood algorithm, revealed that strain F16-60^T^ constituted a monophyletic branch clearly separated from the most closely related species (Figure 1). Maximum-parsimony and neighbour-joining methods resulted in highly similar tree topologies. The low percentages of 16S rRNA gene sequence similarity, as well as its phylogenetic position, clearly support the placement of the new isolate within a new genus and species, separated from the previously described haloarchaeal taxa. The 16S rRNA gene sequence of strain F16-60^T^ was also obtained from its genome, and when compared with that obtained experimentally by PCR, both sequences resulted identical.

**Figure 2:**
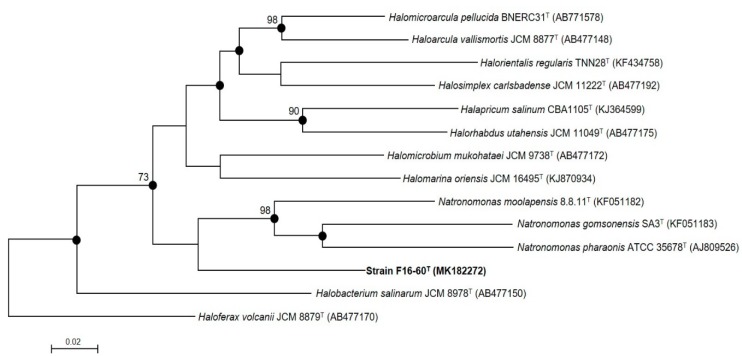
Phylogenetic reconstruction based on the comparison of the *rpoB’* gene of strain F16-60^T^ and related members of the order *Halobacteriales* based on the maximum-likelihood algorithm. Filled circles indicate branches that were also supported by the neighbour-joining algorithm. Bootstrap values higher than 70% are indicated at branch-points. Sequence accession numbers are shown in parentheses. Bar: 0.02 substitutions per nucleotide position. The species *Haloferax volcanii* JCM 8879^T^ was used as an outgroup.

On the other hand, due to the reported limitations on haloarchaea´s phylogeny based on the 16S rRNA gene [54,55], other phylogenetic approaches are recommended for the comparison of haloarchaea. The *rpoB’* gene of strain F16-60^T^ (1848 bp) was also sequenced and compared with those of the most closely related species. The phylogenetic tree based on the *rpoB’* gene sequence using the maximum-likelihood algorithm (Figure 2) clearly showed a distinct monophyletic clade for strain F16-60^T^, with values of similarity in all cases lower than 87.2% with the phylogenetically closest related species. This phylogeny also supports the condition of new genus and species for strain F16-60^T^. A similar topology was obtained by the neighbour-joining algorithm.

**Figure 3:**
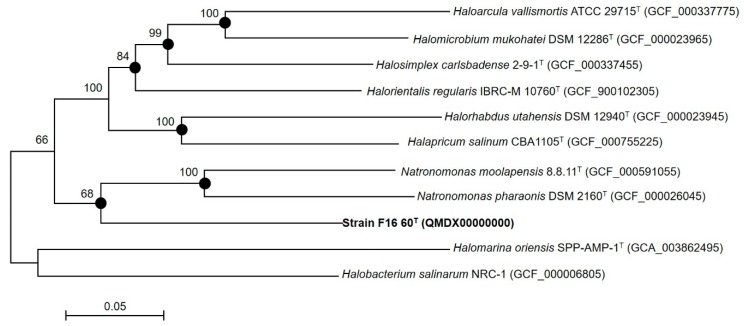
Maximum-likelihood core gene phylogenetic tree including 11 genomes of members of the order *Halobacteriales*. This tree was based on the Jukes–Cantor distance calculated from the alignment of 170 shared orthologous single-copy genes of these genomes. Filled circles indicate branches that were also supported by the neighbour-joining algorithm. Bootstrap values higher than 60% are indicated at branch-points. Bar: 0.05 substitutions per nucleotide position.

To carry out a complete phylogenic analysis, a phylogenomic tree based on core orthologous genes was also constructed. The phylogenomic tree reconstruction (Figure 3) based on a total of 170 orthologous genes shared between all genomes under study supports the placement of strain F16-60^T^ within a new genus and species most closely related to species of the genus *Natronomonas* and other genera of the family *Haloarculaceae*, in the order *Halobacteriales*. These studies clearly show that strain F16-60^T^ constitutes a distinct clade clearly separated from the most closely related species, with a bootstrap value of 68%, supporting its classification as a new genus.

**Figure 4:**
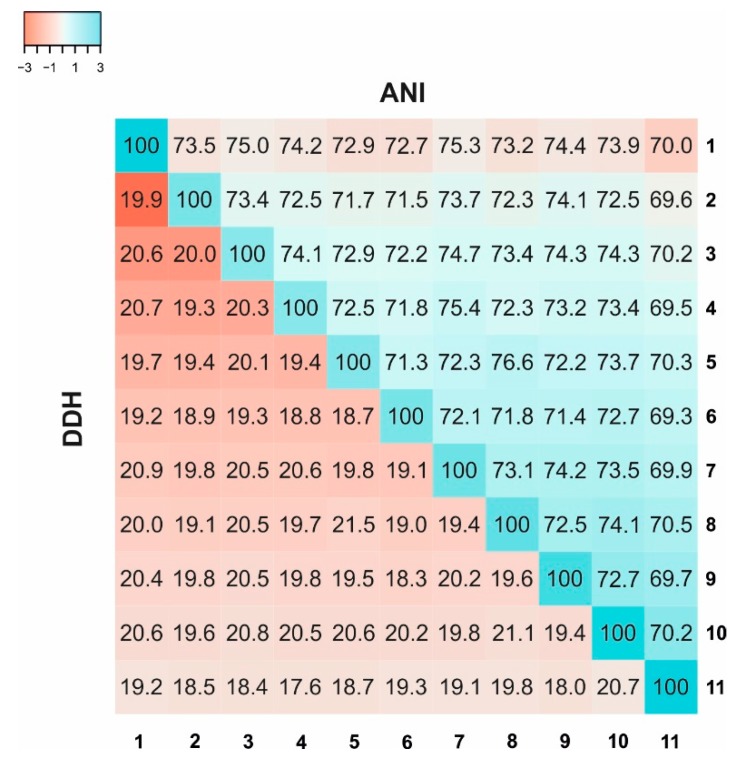
Average Nucleotide Identity (ANI) and in silico DNA–DNA hybridization (DDH) percentages between strain F16-60^T^ and other species and genera from the order *Halobacteriales*. Average Nucleotide Identity (ANI) and in silico DNA–DNA hybridization (DDH) are represented by heat maps, where similarity percentages are represented by the color key histograms on the upper panel. Strains: 1. *Halosimplex carlsbadense* 2-9-1^T^ (GCF_000337455); 2. *Halorhabdus utahensis* DSM 12940^T^ (GCF_000023945); 3. *Halorientalis regularis* IBRC-M 10760^T^ (GCF_900102305); 4. *Haloarcula vallismortis* ATCC 29715^T^ (GCF_000337775); 5. *Natronomonas pharaonis* DSM 2160^T^ (GCF_000026045); 6. *Halobacterium salinarum* NRC-1 (GCF_000006805); 7. *Halomicrobium mukohatei* DSM 12286^T^ (GCF_000023965); 8. *Natronomonas moolapensis* 8.8.11^T^ (GCF_000591055); 9. *Halapricum salinum* CBA 1105^T^ (GCF_000755225); 10. Strain F16-60^T^ (QMDX00000000); 11. *Halomarina oriensis* SPP-AMP-1^T^ (GCA_003862495).

Considering the results obtained by the complete phylogenetic analyses, in order to confirm that strain F16-60^T^ was indeed a new taxon, Average Nucleotide Identity (ANI), Average Amino Acid Identity (AAI), and in silico DNA–DNA hybridization (DDH) of strain F16-60^T^ and other related members of the order *Halobacteriales*, were calculated (Figure 4 and Table 1). Results of the ANI and in silico DDH estimation for strain F16-60^T^ and the related haloarchaea (Figure 4) were in all cases lower than 95% and 70%, respectively, which are the threshold values for species delineation [39,56,57,58]. These results indicate that strain F16-60^T^ constitutes a new taxon at least at the species level, not related at this level to any of the closest phylogenetic neighbours. Additionally, species that share less than 80% ANI (Figure 4), are too divergent to be compared only based on this parameter, and AAI, which provides a more robust resolution, should be used instead [59]. Thus, AAI percentages were also estimated and the results are shown in Table 1. A threshold AAI value of 65% has been established for genus delineation [60,61]. AAI values for strain F16-60^T^ and the most closely related species were in all cases lower than 65% (Table 1), confirming its condition of a new genus.

According to the phylogenetic analysis and phylogenomic analyses, and the ANI, AAI, and in silico DDH data, strain F16-60^T^ constitutes a new genus and species, for which we propose the new designations of *Haloglomus* gen. nov., and *Haloglomus irregulare* sp. nov., respectively. Since the most closely related species of this new taxon belongs to the family *Haloarculaceae*, including *Haloarcula vallismortis*, type species of *Haloarcula*, we suggest that this new genus should be also classified within this family member of the order *Halobacteriales*, within the class *Halobacteria*.

### 3.2. Chemotaxonomic Characterization

To describe strain F16-60^T^, the complete chemotaxonomic characterization of this strain was carried out. The total amount of lipids was extracted and compared with those from closely related neighbours, *Natronomonas pharaonis* CECT 4578^T^ and *Natronomonas moolapensis* CECT 7526^T^ and the reference strains *Halobacterium salinarum* DSM 3754^T^ and *Halorubrum saccharovorum* DSM 1137^T^. The major polar lipids of strain F16-60^T^ were phosphatidylglycerol (PG), phosphatidylglycerol phosphate methyl ester (PGP-Me), phosphatidylglycerol sulfate (PGS), and one glycolipid chromatographically identical to sulphated mannosyl glucosyl diether (S-DGD-1) (Figures S1 and S2). 

In comparison with species of the genus *Natronomonas*, it is remarkable that the absence of biphosphatidylglycerol (BPG) and sulfated triglycosyl diphytanyl archaeol ester linked to phosphatidic acid (S-TGD-1-PA) in strain F16-60^T^, which are present in *Natronomonas moolapensis* CECT 7526^T^ (Figures S1 and S2). 

### 3.3. Phenotypic Characterization

Cells of strain F16-60^T^, were Gram-stain-negative, irregular shaped (0.6 x 3.0 µm) and non-motile (Figure S3). Colonies are red-pigmented with a diameter of 0.5 mm in a modified DBCM2 medium after 20 days of incubation at 37 °C. Strain F16-60^T^ was strictly aerobic, able to grow over a small range of NaCl concentrations, from 20% to 35% (*w*/*v*) (optimum 30% (*w*/*v*) NaCl) and over a pH range from 6.5 to 9.0 (optimum 7.5), confirming its condition of extremely halophilic and neutrophilic archaeon and thus belonging to the non-alkaliphilic haloarchaeal group. Mg^2+^ was not required for growth. Strain F16-60^T^ was catalase positive, oxidase negative, and able to reduce nitrate and nitrite without gas production. Voges–Proskauer, indole production, Simmons´s citrate, and urease tests were negative. Other phenotypic characteristics, range and optimum temperature, pH and NaCl concentrations for growth, hydrolysis of different compounds, and utilization of several substrates as single source of carbon and energy are detailed in the species description and in Table 2. 

### 3.4. Genomic Characteristics

The draft genome of strain F16-60^T^ was successfully sequenced and de novo assembled in a total of 54 contigs. The genome size was 4019787 bp and the sequencing depth 340.85X coverage of the entire genome. The DNA G+C content was 68.0 mol%. Additional genomic characteristics are shown in Table 3. All genomic characteristics are in accordance with the Minimal Standards established for the use of genomic data in prokaryotic taxonomy [14].

Additionally, to determine the number of genes shared between strain F16-60^T^ and the two closest related species, *Natronomonas pharaonis* and *Natronomonas moolapensis*, a Venn Diagram was constructed (Figure S4).

### 3.5. Metagenomic Fragment Recruitment Analysis

**Figure 5:**
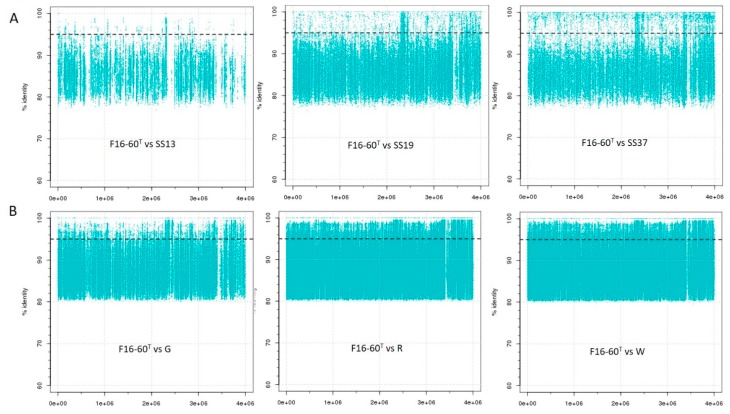
Metagenomic fragment recruitment plots of strain F16-60^T^ against the metagenomic datasets: (**A**) SS13, SS19, and SS37, from Santa Pola saltern; (**B**) G, R, and W from Lake Meyghan (Iran). In each panel the Y-axis represents the identity percentage and X-axis represents the genome length. A restrictive cut-off 95% of nucleotide identity in at least 30 bp of the metagenomic read was used. The black dashed line shows the threshold for the presence of the same species (95% identity). Abbreviations: SS13: Metagenome from Santa Pola saltern (Spain) with 13% salinity; SS19: Metagenome from Santa Pola saltern (Spain) with 19% salinity; SS37: Metagenome from Santa Pola saltern (Spain) with 37% salinity; G: Metagenome from Lake Meyghan (Iran) with 5% salinity; R: Metagenome from Lake Meyghan (Iran) with 18% salinity; W: Metagenome from Lake Meyghan (Iran) with 30% salinity.

To assess the presence in hypersaline habitats from different locations and the geographical distribution of haloarchaeal strains related at species level to strain F16-60^T^, fragment recruitments with several environmental metagenomic datasets (Table S1) were performed. Results are shown in Figure 5 and in Figure S5. 

Figure 5 represents recruitment plots from three different metagenomic datasets obtained from different samples, ordered by salinity gradient, corresponding to two different hypersaline environments, Santa Pola saltern, located in East Spain, and Lake Meyghan in Iran. In both environments, the presence and abundance of strain F16-60^T^ in the metagenomes increases with the salinity, indicating that strain F16-60^T^ is a halophilic archaeon which prefers to inhabit ecosystems with moderate to high salinities.

Additionally, data indicated that even strain F16-60^T^ is not highly abundant in most hypersaline environments studied, it is widely distributed, as it is present in different geographically remote habitats (Figure 5 and Figure S5). On the other hand, the abundance of reads above 95% similarity against SS37, R, W, and S7 metagenomic datasets (Figure 5 and Figure S5), also suggests the existence of additional species of the genus *Haloglomus* or at least closely related, which are present in these habitats but have not yet been isolated. 

### 3.6. Description of *Haloglomus* gen. nov.

*Haloglomus* (Ha.lo.glo´mus. Gr. masc. n. *hals*, *halos* salt; L. neut. n. *glomus* a ball; N.L. neut. n. *Haloglomus*, a salt ball).

Cells are Gram-stain-negative, non-motile, and pleomorphic, curved to irregular shaped. Red-pigmented colonies. Extremely halophilic. Strictly aerobic. Catalase positive but oxidase negative. The polar lipids are phosphatidylglycerol (PG), phosphatidylglycerol phosphate methyl ester (PGP-Me), phosphatidylglycerol sulfate (PGS), and one glycolipid chromatographically identical to sulfated mannosyl glucosyl diether (S-DGD-1).

The type species is *Haloglomus irregulare*. The DNA G + C content is 68.0 mol%. Phylogenetically affiliated to the family *Haloarculaceae*, within the order *Halobacteriales*, and distantly related to the genus *Natronomonas* (≤ 92.9% sequence similarity on the basis of 16S rRNA sequence analysis). The recommended three-letter abbreviation: *Hgl*.

### 3.7. Description of *Haloglomus irregulare* sp. nov.

*Haloglomus irregulare* (ir.re.gu.la´re. L. neut. adj. *irregulare*, irregular cells).

Cells are non-motile, irregular pleomorphic shaped (0.6 × 3 µm) (Figure S3) and Gram-stain negative. Colonies are red pigmented, entire, round, 0.5 mm of diameter in a modified DBCM2 medium after 20 days of incubation at 37 °C. Extremely halophilic and neutrophilic. Cells require 20%–35% (*w*/*v*) NaCl, grow at pH 6.5–9.0, and at 30–45 °C. Mg^2+^ is not required for growth. Optimal growth occurs at 30% (*w*/*v*) NaCl, pH 7.5 and 37 °C. Strictly aerobic. Anaerobic growth does not occur in the presence of arginine, KNO_3_ or DMSO. Catalase positive and oxidase negative. Positive for nitrate and nitrite reduction without gas production, gelatin hydrolysis and weakly for methyl red test, but negative for Voges–Proskauer, indole production, citrate, urease and H_2_S production. Does not hydrolyse DNA or Tween 80. Acid is produced from D-arabinose, D-cellobiose, citruline, fructose, D-glucose, D-ribose and D-xylose, but not from D-amygdalin, arbutine, D-dulcitol, D-galactose, glycerol, maltose, D-mannitol, D-mannose, D-melezitose, D-raffinose, sucrose, sorbitol, D-trehalose, or xylitol. Utilizes D-glucose, D-melibiose, D-raffinose, L-alanine, L-cysteine, and pyruvate as sole carbon and energy source, but does not utilize D-arabinose, D-cellobiose, fructose, D-galactose, lactose, maltose, D-mannose, D-ribose, sucrose, D-trehalose, D-xylose, D-melezitose, salicin, buthanol, dulcitol, ethanol, glycerol, D-mannitol, sorbitol, xylitol, methanol, L-arginine, glutamine, L-methionine, L-glycine, L-lysine, isoleucine, valine, benzoate, citrate, formiate, fumarate, propionate, valerate, hippurate, malate, or tartrate. Polar lipids include phosphatidylglycerol (PG), phosphatidylglycerol phosphate methyl ester (PGP-Me), phosphatidylglycerol sulfate (PGS), and one glycolipid chromatographically identical to sulfated mannosyl glucosyl diether (S-DGD-1). The DNA G + C content is 68.0 mol% (genome).

The type strain is F16-60^T^ (= CECT 9635^T^ = JCM 33318^T^), isolated from a marine saltern located in Isla Cristina, Huelva, Spain.

The GenBank/EMBL/DDBJ accession number for the 16S rRNA and *rpoB´* gene sequences of *Haloglomus irregulare* F16-60^T^ are MH424600 and MK182272, respectively, and that of the genome is QMDX00000000.

## 4. Conclusions

Considering that recent studies were carried out on hypersaline environments have brought to light that a huge number of haloarchaea remain uncultured, we designed new strategies to isolate them. During the course of studies at Isla Cristina marine saltern in Spain, a new extremely halophilic and neutrophilic archaeon, designated strain F16-60^T^ was isolated in pure culture and selected for further analyses. Therefore, the taxonomic study, based on a combination of phylogenetic, chemotaxonomic, and phenotypic analysis, as well as the comparative genomic study, supports the classification of strain F16-60^T^ within a separate genus and species, for which we propose the new designation *Haloglomus irregulare* gen. nov., sp. nov. Metagenomic fragment recruitments indicated that this new taxon prefers to inhabit environments with intermediate to high salinities.

## Figures and Tables

**Table 1:** Average Amino Acid Identity (AAI) percentages, highlighted in bold, between strain F16-60^T^ and other species and genera from the order *Halobacteriales*.

AAI Values	1	2	3	4	5	6	7	8	9	10	11
**1**	-	64.0	65.2	65.2	62.8	61.6	65.9	62.4	65.1	**62.5**	61.3
**2**	-	-	64.0	63.9	62.7	60.8	64.4	62.2	66.3	**61.8**	61.1
**3**	-	-	-	65.3	64.0	62.1	65.6	63.8	65.3	**64.0**	62.9
**4**	-	-	-	-	62.5	61.5	68.7	62.9	65.4	**63.1**	61.7
**5**	-	-	-	-	-	62.7	63.3	73.0	63.2	**64.9**	62.4
**6**	-	-	-	-	-	-	61.9	61.2	61.3	**61.4**	60.6
**7**	-	-	-	-	-	-	-	63.4	65.9	**62.9**	61.9
**8**	-	-	-	-	-	-	-	-	63.0	**64.6**	62.5
**9**	-	-	-	-	-	-	-	-	-	**62.4**	62.0
**10**	-	-	-	-	-	-	-	-	-	-	62.0
**11**	-	-	-	-	-	-	-	-	-	-	-

Strains: 1. *Halosimplex carlsbadense* 2-9-1^T^ (GCF_000337455); 2. *Halorhabdus utahensis* DSM 12940^T^ (GCF_000023945); 3. *Halorientalis regularis* IBRC-M 10760^T^ (GCF_900102305); 4. *Haloarcula vallismortis* ATCC 29715^T^ (GCF_000337775); 5. *Natronomonas pharaonis* DSM 2160^T^ (GCF_000026045); 6. *Halobacterium salinarum* NRC-1 (GCF_000006805); 7. *Halomicrobium mukohatei* DSM 12286^T^ (GCF_000023965); 8. *Natronomonas moolapensis* 8.8.11^T^ (GCF_000591055); 9. *Halapricum salinum* CBA 1105^T^ (GCF_000755225); 10. Strain F16-60^T^ (QMDX00000000); 11. *Halomarina oriensis* SPP-AMP-1^T^ (GCA_003862495).

**Table 2:** Differential characteristics of strain F16-60^T^ and *Natronomonas moolapensis* CECT 7526^T^.

Characteristic	1	2
Morphology	Irregular, pleomorphic	Rods or pleomorphic *
Motility	−	+ *
Cell size (µm)	0.6–3.0	0.7 × 1.7 *
Colony size (mm)	0.5	0.5–1.0 *
Colony-pigmentation	Red	Pink *
NaCl Range (%, *w*/*v*)	25–35	14–36 *
NaCl optimum (%, *w*/*v*)	30	18–20 *
Temperature range for growth (°C)	25–45	25–45 *
pH range	6.5–9.0	5.5–8.5 *
pH optimum	7.5	7–7.5 *
Nitrite reduction	+	−
Hydrolisis of gelatin	+	−
Utilization as sole carbon and energy source of:		
d-glucose	+	−
d-melibiose	+	−
d-raffinose	+	−
Glycerol	−	+
l-cysteine	+	−
l-glycine	−	+
l-lysine	−	+
Isoleucine	−	+
Valine	−	+
Fumarate	−	+
Malate	−	+
Pyruvate	+	−

Strains: 1. *Haloglomus irregulare* F16-60^T^; 2. *Natronomonas moolapensis* CECT 7526^T^. All data from this study, except * which were obtained from the original description [62]. +: Positive; −: negative.

**Table 3:** General features of the sequenced genome.

Feature	Strain F16-60^T^
Size (bp)	4019787
Contigs	54
Completeness (%)	97.6
G + C (mol%)	68.0
N50 (bp)	274728
Total genes	3922
Protein coding genes	3673
rRNA	4
tRNA	53
Accession number	QMDX00000000

## Supplementary Materials

The following are available online at https://www.mdpi.com/2076-2607/8/2/206/s1. Table S1: Features of the different databases from hypersaline habitats used for metagenomic fragment recruitments; Figure S1: High performance thin layer chromatography (HPTLC) of polar lipids extracted from strain F16-60^T^ and some other haloarchaeal species; Figure S2: High performance thin layer chromatography (HPTLC) of phospholipids extracted from strain F16-60^T^ and some other haloarchaeal species; Figure S3: Photomicrograph of cells of strain F16-60^T^ observed under a phase-contrast microscope (1000X, immersion oil), cultured in a liquid medium under optimal conditions; Figure S4: Venn diagram showing the number of genes shared between the genome of strain F16-60^T^ and closest related species *Natronomonas pharaonis* DSM 2160^T^ and *Natronomonas moolapensis* 8.8.11^T^; Figure S5: Metagenomic fragment recruitment plots of strain F16-60^T^ against different metagenomic datasets.
