# Supplementary material for: Haloglomus irregulare gen. nov., sp. nov., a New Halophilic Archaeon Isolated from a Marine Saltern
Source: Microorganisms. 2020 Feb 2;8(2):206. doi: 10.3390/microorganisms8020206 (PMC7074781; doi:10.3390/microorganisms8020206)
Supplement: Supplementary file 1 [file microorganisms-08-00206-s001.pdf]

microorganisms

MDPI

Supplementary Material

Haloglomus irregulare gen. nov., sp. nov., a New Halophilic Archaeon Isolated from a Marine Saltern

Ana Durán-Viseras, Cristina Sánchez-Porro and Antonio Ventosa*

Department of Microbiology and Parasitology, Faculty of Pharmacy, University of Sevilla, 41012 Sevilla, Spain

* Correspondence: ventosa@us.es; Tel.: +34-954556765

Table S1. Features of the different metagenomic databases from hypersaline habitats used for metagenomic fragment recruitments.

Database name	Habitat	Salinity	Accession number	Reference
G	Saline lake, Iran	5 % NaCl	ERS1455389	[1]
SS13	Saltern, Spain	13 % NaCl	SRX328504	[2]
R	Saline lake, Iran	18 % NaCl	ERS1455390	[1]
SS19	Saltern, Spain	19 % NaCl	SRX090228	[3]
IC21	Saltern, Spain	21 % NaCl	SRX352042	[4]
Tyrrell 0.1	Saline lake, Australia	29 % NaCl	SRR5637210	[5]
Tyrrell 0.8	Saline lake, Australia	29 % NaCl	SRR5637211	[5]
S7	Saline lake, Romania	30 % NaCl	SRR8921445	Unpublished
W	Saline lake, Iran	30 % NaCl	ERS1455391	[1]
SS33	Saltern, Spain	33 % NaCl	SRX347883	[2]
Cahuill	Saltern, Chile	34 % NaCl	SRX680116	[6]
SS37	Saltern, Spain	37 % NaCl	SRX090229	[3]
Gujarat	Saline soil, India	ND	ERP005612	[7]
SMO1	Saline soil, Spain	24.0 dS/m	SRR5753725	[8]
SMO2	Saline soil, Spain	54.4 dS/m	SRR5753724	[8]

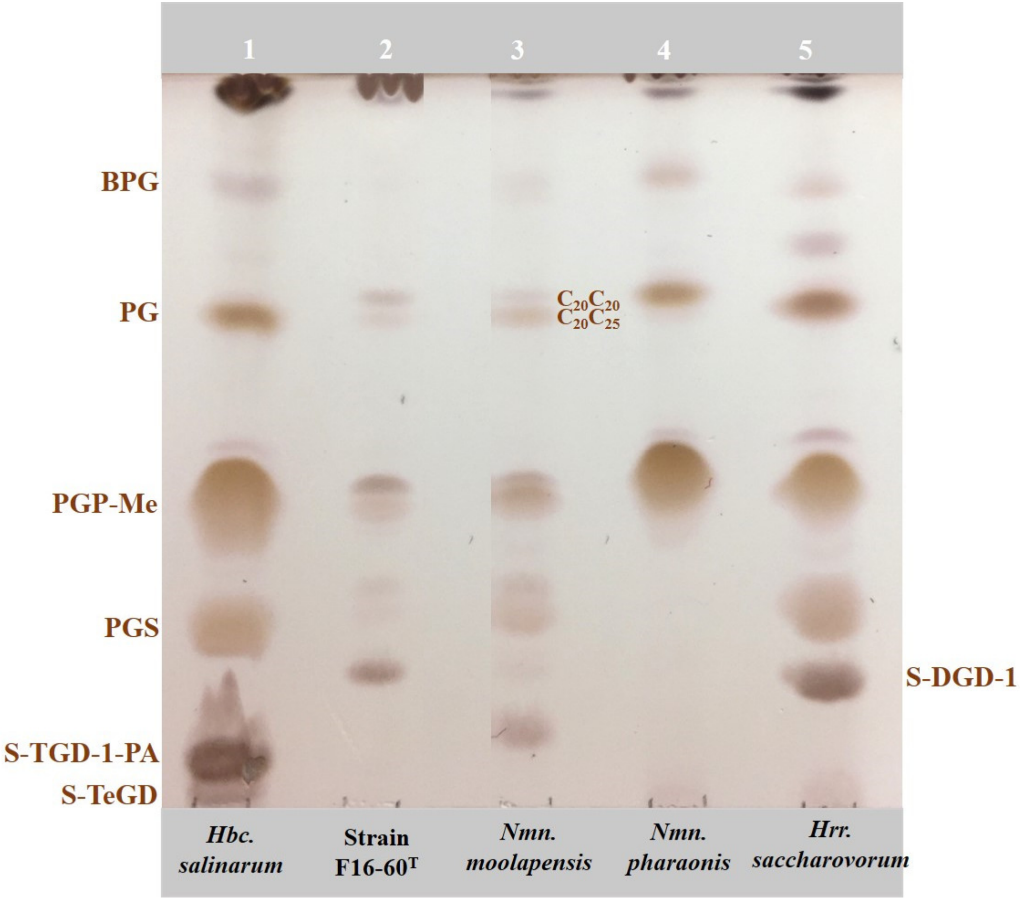

Figure S1. High performance thin layer chromatography (HPTLC) of polar lipids extracted from strain F16-60^{T} and some other haloarchaeal species. The plate was revealed with sulfuric acid 5 % in water, and charred by heating at 160 °C. **Lanes: 1,** *Halobacterium salinarum* DSM 3754^{T}; **2,** *Haloglomus irregulare* gen. nov., sp. nov. (strain F16-60^{T}); **3,** *Natronomonas moolapensis* CECT 7526^{T}; **4,** *Natronomonas pharaonis* CECT 4578^{T}; **5,** *Halorubrum saccharovorum* DSM 1137^{T}.

Abbreviations: BPG, biphosphatidylglycerol; PG, phosphatidylglycerol; PGP-Me, phosphatidylglycerol phosphate methyl ester; PGS, phosphatidylglycerol sulfate; S-DGD-1, sulfated diglycosil diether; S-TGD-1-PA, sulfated triglycosyl diphytanyl archaeol ester linked to phosphatidic acid; S-TeGD, sulfated tetraglycosil diether.

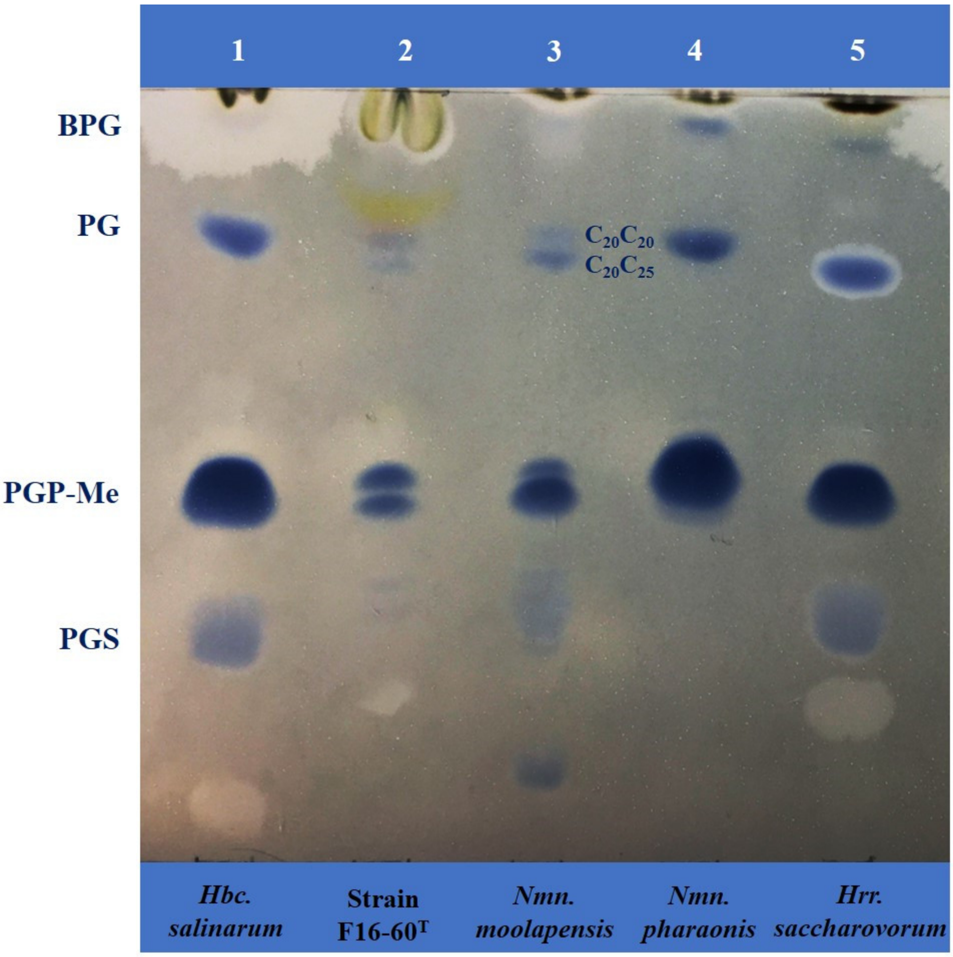

Figure S2. High performance thin layer chromatography (HPTLC) of phospholipids extracted from strain F16-60^T and some other haloarchaeal species. The plate was revealed with molibden blue. **Lanes: 1,** *Halobacterium salinarum* DSM 3754^T; **2,** *Haloglomus irregulare* gen. nov., sp. nov. (strain F16-60^T); **3,** *Natronomonas moolapensis* CECT 7526^T; **4,** *Natronomonas pharaonis* CECT 4578^T; **5,** *Halorubrum saccharovorum* DSM 1137^T.

Abbreviations: BPG, biphosphatidylglycerol; PG, phosphatidylglycerol; PGP-Me, phosphatidylglycerol phosphate methyl ester; PGS, phosphatidylglycerol sulfate; S-DGD-1, sulfated diglycosil diether.

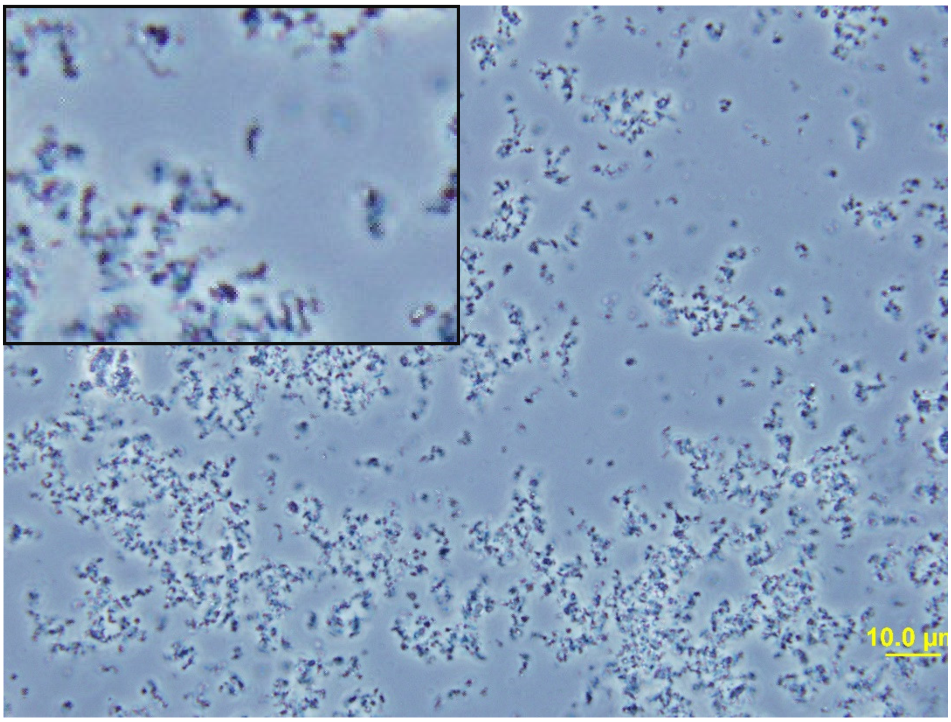

Figure S3. Photomicrograph of cells of strain F16-60^T observed under a phase-contrast microscope (1000X, immersion oil), cultured in liquid medium under optimal conditions. Scale bar, 10 µm.

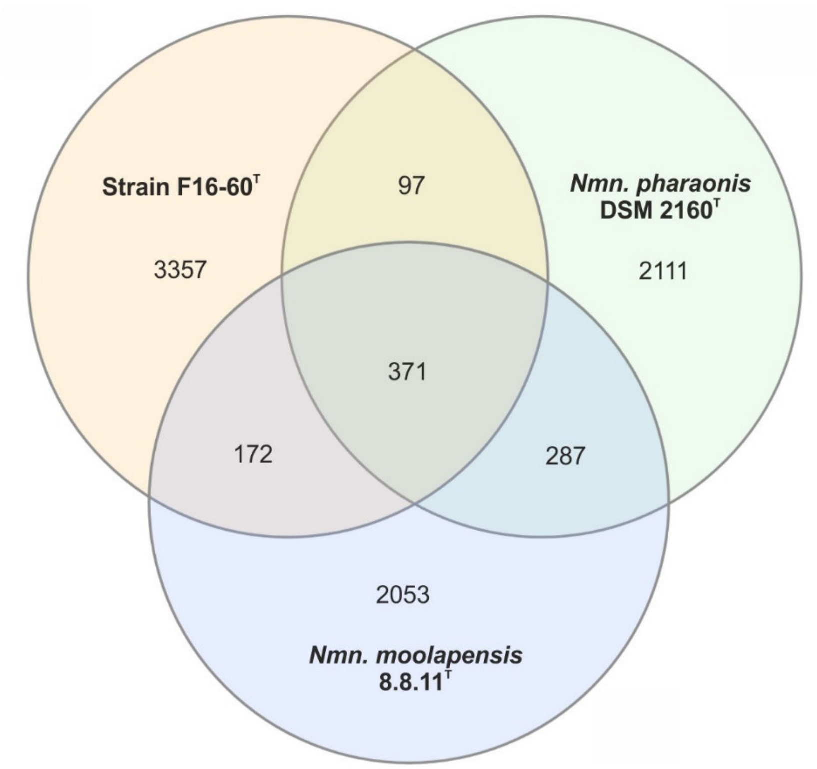

Figure S4. Venn diagram showing the number of genes shared between the genome of strain F16-60^T and closest related species *Natronomonas pharaonis* DSM 2160^T and *Natronomonas moolapensis* 8.8.11^T.

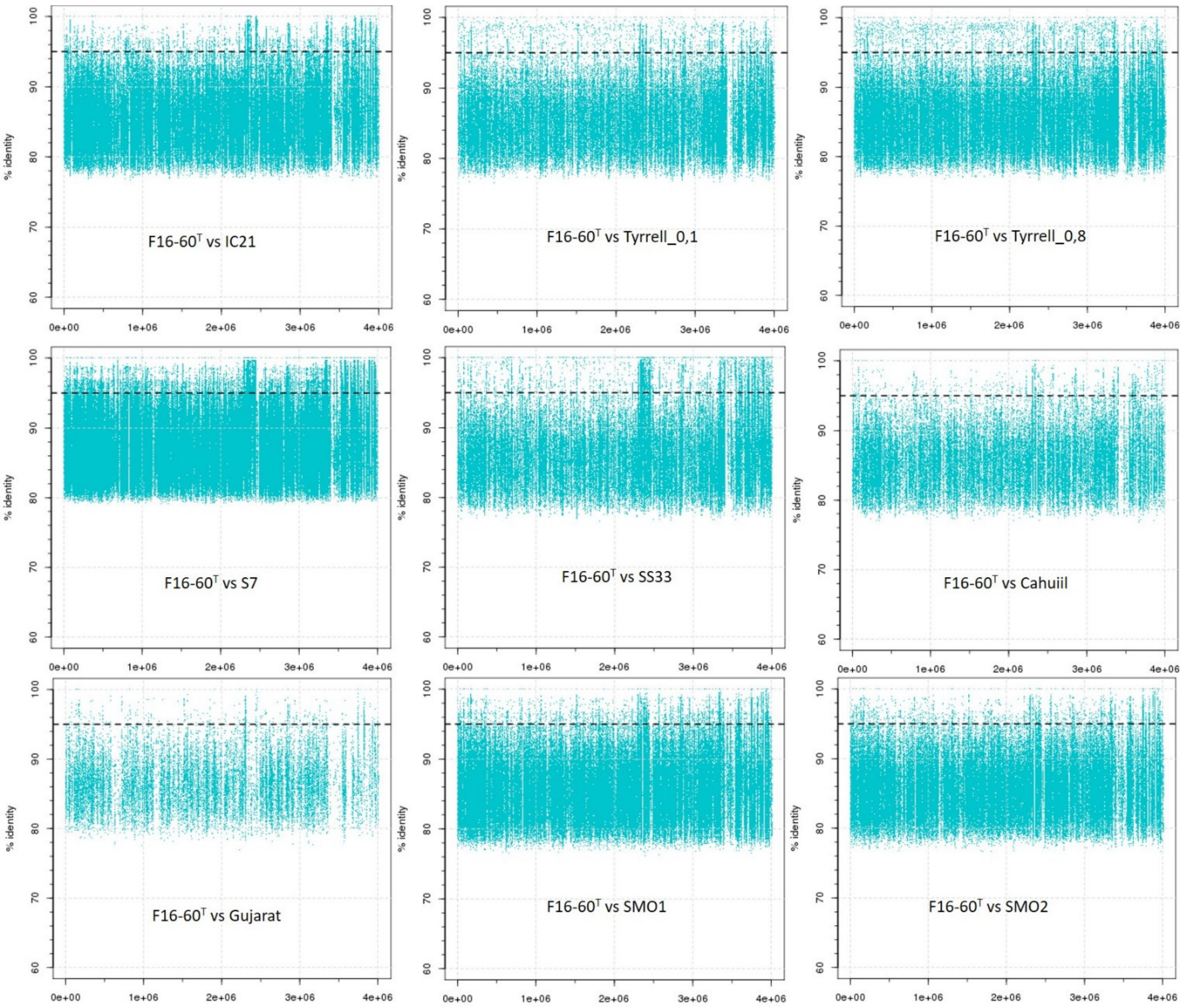

Figure S5. Metagenomic fragment recruitment plots of strain F16-60^{T} against different metagenomic datasets (see Supplementary Table 1). In each panel the Y axis represents the percentage of identity and X axis represents the genome length. A restrictive cut-off 95 % of nucleotide identity in at least 30 bp of the metagenomic read was used. The black dashed line shows the threshold for the presence of the same species (95 % identity).

Abbreviations: IC21 – Metagenome from Isla Cristina solar saltern pond (Spain), 21 % salinity, Tyrrell 0.1 – Metagenome from Lake Tyrrell (Australia), 29 % salinity, Tyrrell 0.8 – Metagenome from Lake Tyrrell (Australia), 29 % salinity, S7 – Metagenome from Fara Fund hypersaline meromictic lake, 30 % salinity, SS33 – Metagenome from Santa Pola solar saltern pond (Spain), 33 % salinity, Cahuill – Metagenome from Cahuil lagoon (Chile), 34 % salinity, Gujarat – Metagenome from Little Rann of Kutch hypersaline soil (India), SMO1 – Metagenome from Marismas del Odiel Salt Marshes hypersaline soil (Spain), 24 mS/cm salinity, SMO2 – Metagenome from Marismas del Odiel Salt Marshes hypersaline soil (Spain), 54 mS/cm salinity.

Supplementary references

1. Naghoni, A.; Emtiazi, G.; Amoozegar, M.A.; Cretoiu, M.S.; Stal, L.J.; Etemadifar, Z.; Shahzadeh Fazeli, S.A.; Bolhuis, H. Microbial diversity in the hypersaline Lake Meyghan, Iran. *Sci. Rep.* **2017**, *7*, 11522.

2. Fernández, A.B.; Ghai, R.; Martin-Cuadrado, A.-B.; Sánchez-Porro, C.; Rodriguez-Valera, F.; Ventosa, A. Prokaryotic taxonomic and metabolic diversity of an intermediate salinity hypersaline habitat assessed by metagenomics. *FEMS Microbiol. Ecol.* **2014**, *88*, 623–635.

3. Ghai, R.; Pašić, L.; Fernández, A.B.; Martin-Cuadrado, A.-B.; Mizuno, C.M.; McMahon, K.D.; Papke, R.T.; Stepanauskas, R.; Rodriguez-Brito, B.; Rohwer, F.; et al. New abundant microbial groups in aquatic hypersaline environments. *Sci. Rep.* **2011**, *1*, 135.

4. Fernández, A.B.; Vera-Gargallo, B.; Sánchez-Porro, C.; Ghai, R.; Papke, R.T.; Rodriguez-Valera, F.; Ventosa, A. Comparison of prokaryotic community structure from Mediterranean and Atlantic saltern concentrator ponds by a metagenomic approach. *Front. Microbiol.* **2014,** *5*, 196.

5. Podell, S.; Emerson, J.B.; Jones, C.M.; Ugalde, J.A.; Welch, S.; Heidelberg, K.B.; Banfield, J.F.; Allen, E.E. Seasonal fluctuations in ionic concentrations drive microbial succession in a hypersaline lake community. *ISME J.* **2014**, *8*, 979–990.

6. Plominsky, A.M.; Delherbe, N.; Ugalde, J.A.; Allen, E.E.; Blanchet, M.; Ikeda, P.; Santibanez, F.; Hanselmann, K.; Ulloa, O.; De la Iglesia, R.; et al. Metagenome sequencing of the microbial community of a solar saltern crystallizer pond at Cahuil lagoon, Chile. *Genome Announc.* **2014**, *2*, e01172-14.

7. Patel, R.; Mevada, V.; Prajapati, D.; Dudhagara, P.; Koringa, P.; Joshi, C.G. Metagenomic sequence of saline desert microbiota from wild ass sanctuary, Little Rann of Kutch, Gujarat, India. *Genomics Data* **2015**, *3*, 137–139.

8. Vera-Gargallo, B.; Ventosa, A. Metagenomic insights into the phylogenetic and metabolic diversity of the prokaryotic community dwelling in hypersaline soils from the Odiel Saltmarshes (SW Spain). *Genes* **2018**, *9*, 152.

© 2020 by the authors. Submitted for possible open access publication under the terms and conditions of the Creative Commons Attribution (CC BY) license (http://creativecommons.org/licenses/by/4.0/).
